# Cognitive Control of Working Memory: A Model-Based Approach

**DOI:** 10.3390/brainsci11060721

**Published:** 2021-05-28

**Authors:** Russell J. Boag, Niek Stevenson, Roel van Dooren, Anne C. Trutti, Zsuzsika Sjoerds, Birte U. Forstmann

**Affiliations:** 1 Integrative Model-Based Cognitive Neuroscience Research Unit, University of Amsterdam, 1018 WS Amsterdam, The Netherlands; niek.stevenson@gmail.com (N.S.); a.c.trutti@uva.nl (A.C.T.); buforstmann@gmail.com (B.U.F.); 2 Cognitive Psychology Unit, Institute of Psychology & Leiden Institute for Brain and Cognition, Leiden University, 2300 RC Leiden, The Netherlands; r.van.dooren@fsw.leidenuniv.nl (R.v.D.); z.sjoerds@fsw.leidenuniv.nl (Z.S.)

**Keywords:** working memory, cognitive control, reference-back task, sequential sampling, diffusion decision model

## Abstract

Working memory (WM)-based decision making depends on a number of cognitive control processes that control the flow of information into and out of WM and ensure that only relevant information is held active in WM’s limited-capacity store. Although necessary for successful decision making, recent work has shown that these control processes impose performance costs on both the speed and accuracy of WM-based decisions. Using the reference-back task as a benchmark measure of WM control, we conducted evidence accumulation modeling to test several competing explanations for six benchmark empirical performance costs. Costs were driven by a combination of processes running outside of the decision stage (longer non-decision time) and inhibition of the prepotent response (lower drift rates) in trials requiring WM control. Individuals also set more cautious response thresholds when expecting to update WM with new information versus maintain existing information. We discuss the promise of this approach for understanding cognitive control in WM-based decision making.

## 1. Introduction

A central problem faced by working memory (WM) is that of managing the trade-off between stability—maintaining stable WM representations against interference—and flexibility—keeping WM up to date when goals and task demands change [1,2,3,4,5]. To solve this problem, WM relies on a number of cognitive control processes that control the flow of information into and out of WM and ensure that only relevant information occupies WM’s limited-capacity store [6,7,8,9,10,11,12,13,14,15]. According to prominent neurocomputational theory, the primary mechanism for accomplishing this is an information gate that controls access to WM [16,17,18]. When closed, the gate prevents new information from entering WM, which allows its contents to be maintained in a stable state in the face of distracting or irrelevant information. When open, the gate allows new information into WM (and old information out), which enables WM to remain up to date with information relevant to current goals and task demands [19,20].

Although necessary for successful WM, recent work with the novel reference-back paradigm has shown that these processes impose substantial performance costs on both the speed and accuracy of WM-based decisions [11,12,14,21,22,23,24,25]. However, less is known about how such costs arise out of latent decision processes (e.g., processing rate, time outside of the decision stage, response threshold settings) as instantiated in leading computational theories of WM-based decision making [26,27]. Here, we apply the diffusion decision model (DDM) [26,28] to data from 150 participants to test between several competing explanations for performance costs observed in the reference-back task. In doing so, we provide the first comprehensive computational account of reference-back task performance in terms of underlying cognitive processes. Before explaining our modeling approach and the theoretical framework for understanding these costs, we first introduce the reference-back paradigm in detail and describe the benchmark empirical phenomena we seek to explain.

### 1.1. Measuring WM Control Processes with the Reference-Back Paradigm

The reference-back task is a modified *n*-back task that measures the behavioral costs associated with performing several cognitive control operations involved in WM-based decision making. Unlike the traditional *n*-back, in which multiple operations are confounded on each trial [11], the reference-back allows costs specific to each control process (e.g., updating and gating) to be decomposed by contrasting different trial types (described in Table 1). This feature of the reference-back paradigm has led to important insights into WM control processes, such as the effects of dopaminergic drugs on WM control, and identifying the neural basis of specific WM control processes [21,22,23,24,25].

**Figure 1:**
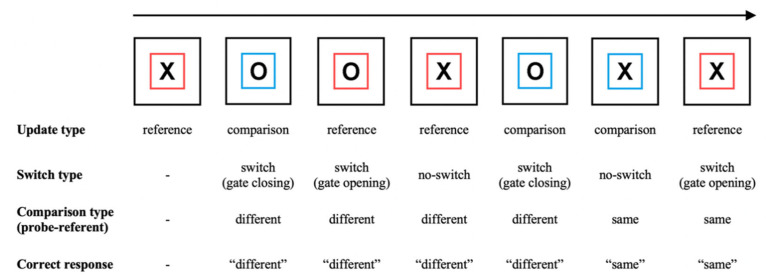
Illustration of the reference-back task. In each trial, subjects indicate whether the probe stimulus (‘X’ or ‘O’) is the same as or different to the stimulus in the most recent red frame (the WM referent). In reference (red frame) trials, subjects must update WM with the currently displayed probe item. In comparison (blue frame) trials, subjects make the ‘same/different’ decision but do not update WM. Comparing behavior (e.g., accuracy, RT) between different trial types measures the costs of several WM control processes (see the main text and Table 1 for details).

Performing the reference-back task involves holding one of two stimuli (an ‘X’ or an ‘O’) in WM while deciding whether a series of probe stimuli are the same as or different to the item currently held in WM (called the reference item or referent). Probe stimuli may either match the referent, requiring a response of ‘same’, or mismatch the referent, requiring a response of ‘different’. Crucially, a colored frame around each probe stimulus cues the subject to either update or maintain the WM referent to which they are comparing each probe. In reference trials, the subject replaces (i.e., updates) their referent with the probe item, which becomes the new referent. In comparison trials, the subject maintains their referent and does not replace it with the probe item. Trials also differ in terms of whether they require switching between updating and maintenance: in switch trials, the update cue (i.e., the frame color) differs from that of the previous trial. In no-switch trials, the update cue is the same as in the previous trial. An illustration of a typical sequence of trials and the resulting updating, switching, and matching requirements is shown in Figure 1.

Importantly, by the logic of subtraction [29], contrasting different trial types (e.g., reference vs. comparison, switch vs. no-switch) provides measures of six cognitive control processes that support WM, and which constitute the effects of interest in typical studies of reference-back performance. These ‘canonical’ measures and their derivations are presented in Table 1. To summarize, three contrasts measure the updating cost (of updating vs. not updating WM), the comparison cost (of processing probe–referent mismatches vs. probe–referent matches), and the switching cost (of switching vs. not switching between updating and maintenance modes). Two gating effects can be derived that decompose switching into costs specific to gate opening (reference/switch vs. reference/no-switch) and gate closing (comparison/switch vs. comparison/no-switch). Finally, the interaction effect of updating (reference/comparison) with the comparison type (same/different) measures substitution—the cost specific to updating WM with a new item (as opposed to refreshing the existing referent).

The benchmark behavioral finding from the reference-back literature is that each cost measure is associated with poorer accuracy (i.e., more frequent errors) and slower RT [11,12,14,21,22,23,24,25]. However, we currently do not have any idea about the underlying cognitive mechanisms that give rise to these costs. In typical reference-back studies, costs are assumed to reflect the time taken for cognitive control and WM operations (e.g., updating, gating, mode switching) to run outside of the probe–referent comparison process. Under this interpretation, trials requiring additional WM control processes should produce an additive shift in RT distributions relative to trials that do not require such processes. However, we note that this ‘additive shift’ hypothesis would not predict the changes in accuracy observed in empirical data. Another possibility is that additional processes modulate or interfere with the probe–referent decision. For example, the signal to update may trigger reactive control processes [30] that inhibit the preparation and execution of prepotent responses (e.g., reactive inhibition in memory-based decision making [31,32,33]; response inhibition in go/no-go and stop-signal tasks [34]). Reactive control refers to event-triggered cognitive control deployed retroactively (i.e., after detecting the critical event) to influence processing “only as needed, in a just-in-time manner” ([30], p. 2). Under this interpretation, both RT and accuracy costs would stem from inhibitory input reducing the speed or efficiency with which the prepotent decision (i.e., the probe–referent comparison) is processed. Finally, it is also possible that certain costs may stem from more strategic top-down adjustments to decision making, such as participants setting stricter response criteria or adopting certain response biases. In order to explore these possibilities, we turned to one of the most successful computational models of memory-based decision making, the DDM [26] (for reviews, see [27,35,36,37]).

### 1.2. The Diffusion Decision Model

**Figure 2:**
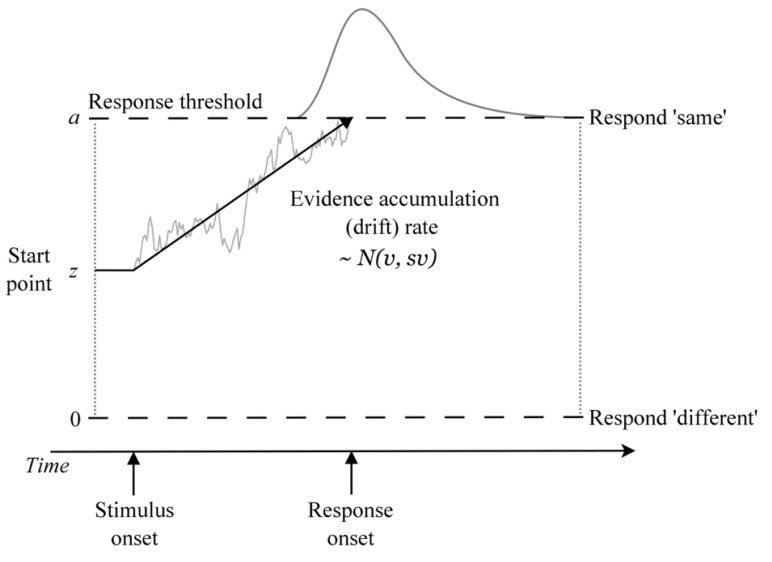
Illustration of the DDM as applied to the reference-back task. Within a trial, the evidence about whether the probe stimulus matches the WM referent is accumulated with mean drift rate *v* until a response threshold is reached, triggering the corresponding response (at the time labeled ‘response onset’). Response time is the time it takes to reach a threshold (decision time) plus an intercept term representing non-decision time processes that occur outside of the decision stage.

The DDM treats decision making as an accumulation-to-threshold process in which noise-perturbed samples of evidence (from the stimulus or its memory trace) are accumulated until a threshold amount is reached, which triggers a response (Figure 2). The DDM provides a full quantitative account of both RT distributions and the accuracy of decisions and accounts for a range of benchmark empirical phenomena that occur in simple decision tasks, including interactions between the speed of correct and error responses, trade-offs between speed and accuracy, response biases, and differences in the duration of processes occurring outside of the decision stage [37]. The DDM has led to important insights into cognition in a wide range of choice tasks, including perceptual-, memory-, and value-based decisions (for a review, see [27]).

The DDM explains effects in terms of model parameters representing the underlying cognitive processes that give rise to behavior. The most important among these are the drift rate, non-decision time, and threshold parameters.

Drift rate (*v*) measures the rate of evidence accumulation, which reflects the quality or signal strength of the evidence driving the decision (analogous to *d*-prime in signal detection theory [38]). In memory-based decision making, drift rate represents the strength of the match between the probe and the item(s) in memory. The original DDM [26] was designed to model the Sternberg task [39], in which subjects decide whether a probe stimulus was present in a previously memorized list. Decisions in this task involve comparing the perceptual stimulus with the (noisy) memory trace of each list item until a match is found. When the memorized list contains only one item, this comparison process is the same as that of the reference-back task (i.e., does the probe match the current item in memory?). Drift rate is thus sensitive to the strength/quality of memory representations [26], as well as global processing demands placed on the cognitive system, such as task difficulty, memory load, and other concurrent processing demands (to the extent that concurrent processes compete for the same cognitive resources [31,32,40]). Drift rates are also a locus of reactive inhibitory control [30], in which critical events (e.g., the need to update WM or task switch) trigger inhibition of prepotent response drift rates [31,32,33]. The effect of reducing drift rate is to produce slower and less accurate responses (and flatter, more skewed RT distributions).

Non-decision time (*t*_0_) accounts for processes that occur outside of the decision (i.e., evidence accumulation) stage. In simple decision tasks, non-decision time typically encompasses processes such as early perceptual encoding and motor response execution [37]. In more complex tasks, non-decision time may be sensitive to additional processes occurring prior to evidence accumulation, such as pre-decisional visual search, attention/task switching, and information gating [41,42,43,44]. In the context of the reference-back task, we assume that WM control processes occur outside of the decision stage and thus should be reflected in non-decision time. The effect of increasing non-decision time is to produce an additive shift in RT distributions along the time axis without affecting accuracy.

The threshold parameter (*a*) represents the amount of evidence required to trigger a response and thus provides a measure of response caution. Thresholds are the locus of cognitive control processes that control speed–accuracy trade-off and response caution settings [28] (e.g., low threshold = fast, error-prone responses; high threshold = slow, accurate responses [45,46]). Individuals often adjust thresholds in order to meet particular goals and contextual requirements (e.g., treating one type of trial more cautiously than another to avoid responding prematurely [47,48]). Setting higher thresholds produces slower and more accurate responses.

The DDM also includes several parameters that are not of immediate theoretical interest, but which improve the model’s ability to fit data. Starting point (*z*) accounts for biases in where the evidence accumulation process begins relative to the thresholds (i.e., response biases), and a number of variability parameters control the amount of trial-to-trial variability in starting point (*sz*) and drift rate (*sv*). In this study, our primary theoretical interest is in the drift rate, non-decision time, and threshold parameters and their contributions to performance on the reference-back task.

### 1.3. Current Study

In the current study, we use the DDM as a measurement model to test several competing explanations for performance costs observed on the reference-back task. Stated in terms of DDM parameters, the cost of WM control processes may be reflected in longer non-decision time (consistent with running extra processes outside of the decision stage), reduced drift rate (consistent with inhibited processing), or both. In addition, individuals may apply different thresholds when cued to update WM versus when cued to maintain WM. To foreshadow our results, our best model indicated that costs were driven by a combination of lower drift rates and longer non-decision times in trials requiring WM control. Individuals also used cognitive control to set more cautious response thresholds when cued to update WM with new information.

## 2. Materials and Methods

### 2.1. Participants

One hundred and fifty participants, recruited from the subject pools of the Institute of Psychology, Leiden University (sample 1: *n* = 89, mean age = 19.11, range 17–26, 82.0% female) and the Department of Psychology, University of Amsterdam (sample 2: *n* = 61, mean age = 19.48, range 17–34, 88.5% female), participated for course credit. All participants had normal or corrected-to-normal vision and gave online written informed consent prior to the experiment onset. The studies were approved by the local ethics committees. The data obtained in sample 1 were part of a larger research project performed at Leiden University. For the current manuscript, only the reference-back data were extracted. The total sample of 150 was the result of pooling two participant groups whose data was collected approximately 6 months apart. Both groups performed the same experimental task under similar conditions and analyses conducted on each group separately produced the same results as those conducted on the pooled sample, both in terms of behavior (i.e., differences in accuracy and RT between conditions) and parameter effects arising in our cognitive model-based analyses. We thus present only the results of the pooled analysis.

### 2.2. Stimuli and Procedure

The reference-back task was coded in JavaScript using the *jsPsych* library [49] and hosted online via the university websites. Each trial began with a blank screen (1 s), followed by a fixation cross (1 s). A probe stimulus (X or O) enclosed in either a red or blue frame was then presented until the subject pressed a response key, followed by another blank screen (0.5 s). The first trial of a block was always a red-framed item in order to provide an initial WM referent and did not require a response. The stimulus and frame color on the remaining trials were randomly sampled with equal probability, meaning that upcoming memory operations could not be predicted prior to the onset of the trial. Subjects thus had to engage cognitive control reactively (i.e., after trial onset), as opposed to proactively (i.e., before the trial onset) [30]. On each trial, subjects were required to make a keyboard response to indicate whether the probe stimulus was the same as or different to the stimulus in the most recent red frame (i.e., to compare the probe to the WM referent). In addition, they were instructed to update their referent with the probe stimulus in reference (red frame) trials and maintain their existing WM referent in comparison (blue frame) trials (see Figure 1). Subjects were told to respond as quickly and accurately as possible. Handedness of the response key arrangement was counterbalanced between subjects. In sample 1, after 12 practice trials, each subject performed 512 trials across four blocks. In sample 2, each subject completed approximately 386 experimental trials across two sessions, with 34 practice trials before session one and 17 before session two.

### 2.3. Analysis Methods

Our primary analysis involved fitting hierarchical Bayesian DDMs to reference-back choice-RT data, assessing model fit, and performing posterior inference on parameter effects. For model fitting, we used the Dynamic Models of Choice (DMC) software [50], which allows for fully Bayesian hierarchical modeling and parameter estimation with the DDM. Details of the fitting procedure and sampling algorithm are provided in Appendix B. We also used DMC to conduct a parameter recovery study, in which we assessed the model’s ability to recover known parameter values from simulated data. The simulation procedure and a visualization of parameter recovery are presented in Appendix C. Conventional statistical analyses and significance tests of behavioral effects were performed in *R* [51] using the *lme4* [52], *lsr* [53], and *car* [54] packages.

## 3. Results

### 3.1. Conventional Analyses

We first report conventional statistical analyses to check whether the different trial types and cost measures in the reference-back task had the expected effects on accuracy and mean RT. We use linear mixed models to test the statistical significance of updating (no-switch/reference, no-switch/comparison), mode-switching (switch, no-switch), match/comparison type (no-switch/same, no-switch/different), gate opening (reference/switch, reference/no-switch), gate closing (comparison/switch, comparison/no-switch), and substitution (interaction of updating with match/comparison type) on mean RT and accuracy. We used a generalized linear mixed model with a probit link function to model accuracy and a general linear mixed model with Gaussian link function to model mean RT. Each model contained the updating, comparison, and switching factors and their interactions as fixed effects and the subject as a random effect. Our criterion for significance was set at 0.05. The results of these tests are tabulated in Appendix A (Table A1 and Table A2). We excluded the first trial from each block (which did not require a response) and excluded RTs faster than 150 ms and slower than 3 s as outliers (~1% of the data).

**Figure 3:**
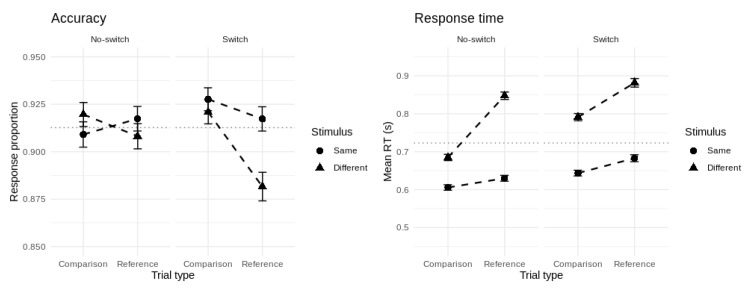
Group-averaged accuracy (**left panel**) and mean RT (**right panel**) for each cell of the experimental design. Error bars represent 95% confidence intervals.

Figure 3 shows the group-averaged accuracy and mean RT for each cell of the experimental design. Overall accuracy on the task was 91.3% and overall mean RT was 723 ms. Correct responses were faster on average than errors (∆ = 94 ms, *t* = 7.29, *df* = 149, *p* < 0.001, *d* = 0.60). Our probit model of accuracy revealed significant main effects of updating and match/comparison type, and no effect of switch type. All two-way interactions between these predictors were significant but their three-way interaction term was not. For our Gaussian model of mean RT, all one-, two-, and three-way terms were significant. To further unpack these effects, we tested the significance of the six canonical reference-back cost contrasts.

Updating: Repeated reference trials produced slower (∆ = 91 ms, *t* = 15.57, *df* = 149, *p* < 0.001, *d* = 1.27) but not less accurate (*t* = 0.53, *df* = 149, *p* = 0.59) responses than repeated comparison trials. This reflects the unique cost of updating versus maintaining the contents of WM.

Mode switching: Switch trials produced slower (∆ = 59 ms, *t* = 13.18, *df* = 149, *p* < 0.001, *d* = 1.08) but not less accurate (*t* = 0.73, *df* = 149, *p* = 0.47) responses than no-switch trials. This reflects the cost of switching between updating and maintenance mode (or vice-versa) compared with remaining in the mode of the previous trial.

Comparison: Stimuli that were different to the most recent reference item produced slower (∆ = 144 ms, *t* = 19.94, *df* = 149, *p* < 0.001, *d* = 1.63) but not less accurate (*t* = 0.15, *df* = 149, *p* = 0.88) responses than stimuli that were the same as the most recent reference item. This reflects the cost of processing stimuli that mismatch the current contents of WM.

Gate opening: Switching from comparison to reference trials produced slower (∆ = 45 ms, *t* = 7.60, *df* = 149, *p* < 0.001, *d* = 0.62) and less accurate (∆ = 1.3%, *t* = 3.54, *df* = 149, *p* < 0.001, *d* = 0.29) responses compared with repeated reference trials. This reflects the cost of opening the gate to WM.

Gate closing: Switching from reference to comparison trials produced slower (∆ = 72 ms, *t* = 13.25, *df* = 149, *p* < 0.001, *d* = 1.08) and slightly more accurate (∆ = 0.9%, *t* = 2.52, *df* = 149, *p* = 0.013, *d* = 0.21) responses compared with repeated comparison trials. This represents the cost of closing the gate to WM.

Substitution: The cost of updating a new versus a repeated item into WM was larger than the cost of comparing (without updating) a new versus a repeated item (Mean RT diff. = 90 ms, *t* = 11.46, *df* = 149, *p* < 0.001, *d* = 0.94, Mean accuracy diff. = 2.5%, *t* = 3.44, *df* = 149, *p* < 0.001, *d* = 0.28). This interaction of updating (reference, comparison) with stimulus type (same, different) represents the cost of substituting a new item into WM.

**Figure 4:**
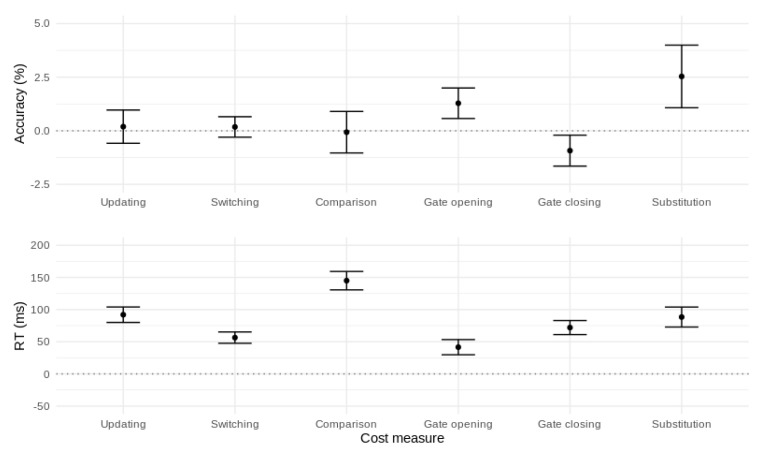
Performance costs affecting accuracy (**top panel**) and mean RT (**bottom panel**) for the canonical reference-back contrasts. Error bars represent 95% confidence intervals.

To summarize, the canonical reference-back cost measures were all associated with slower RT and, for gate opening and substitution, lower accuracy. In addition, the larger RT cost for gate closing versus gate opening replicates the asymmetric gating costs found in previous work [7,11,12,14,21]. These effects are illustrated in Figure 4.

### 3.2. Diffusion Decision Model Analysis

To explore the latent cognitive processes that give rise to the accuracy and RT costs outlined above, we applied the most prominent computational cognitive model of decision-making, the DDM [26,27], to our choice-RT data. Our primary aim in applying the DDM to the reference-back task was to determine the extent to which costs observed at the behavioral level are explained by different DDM parameters. We were particularly interested in establishing whether costs are explained by WM control processes that operate outside of the decision-making stage (i.e., non-decision time effects) or whether control processes affect the accumulation of evidence during decision-making (drift rate effects). In addition, we thought it plausible that subjects might use volitional ‘top-down’ control to adjust their response thresholds in response to the updating cue (i.e., different thresholds for updating vs. maintenance modes). Our starting point was thus a DDM in which drift rate and non-decision time could vary over every design cell and with different thresholds for reference and comparison trials. Prior distributions on model parameters are given in Appendix B. We compared this top model to several constrained variants in which either the drift rate, non-decision time, or threshold effects were removed (i.e., held fixed across design cells). Each model also included one starting point, one starting point variability, and one drift rate variability parameter. Drift criterion and non-decision time variability were fixed at zero to satisfy the scaling constraint of evidence accumulation models [55]. The results of model selection are shown in Table 2.

We used the deviance information criterion (DIC) [56] to measure the relative quality of each model, accounting for both goodness of fit and model complexity (number of parameters). The preferred individual-level model (i.e., with the lowest DIC) for the largest proportion of subjects (37.3%) was the fully flexible top model, suggesting that drift rate, non-decision time, and threshold effects each play a role in explaining performance and costs in the reference-back task. The next most preferred model (30.7%) had the threshold effect removed but still retained both fully flexible drift rates and non-decision times in order to explain the RT and accuracy cost effects. Smaller proportions of subjects were better explained by models with either the drift rate (23.3%) or non-decision time (8.7%) effects removed. We then fit a fully hierarchical version of the top model to obtain group-level and subject-level posterior distributions of each model parameter. Accounting for hierarchical (group) structure imposes further constraint on the model by ‘shrinking’ subject-level estimates towards the group average and thus makes posterior inference on the resulting parameter estimates more conservative. Except where indicated, the remaining analyses will focus on the fits and parameter effects of this hierarchical top model.

### 3.3. Model Fit and Parameter Recovery

**Figure 5:**
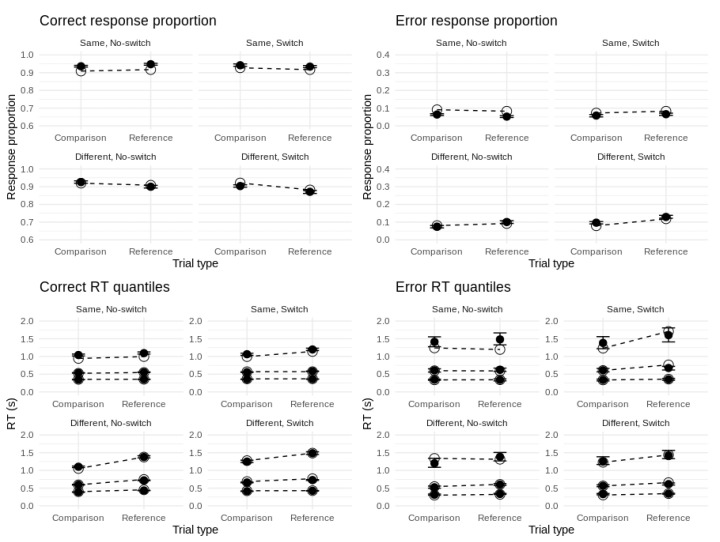
Fits of top model to behavioral data. Top panels: Fits to correct and error proportions. Bottom panels: Fits to the 0.1, 0.5, and 0.9 quantiles of correct and error RT distributions. Unfilled circles represent data effects. Filled circles represent posterior model predictions with 95% credible intervals.

To evaluate how closely the model fits the data, we sampled 100 posterior predictions of choice-RT for each subject and then took the average over all subjects. As Figure 5 shows, the model provides close fits to response proportions and RT distributions for each response type and design cell. We note a slight tendency to overpredict accuracy for responses to ‘same’ stimuli (i.e., probe–referent matches) as well as some minor misfit in the tails of error RTs for ‘same’ stimuli. This is likely due to the high accuracy and relatively small proportion of error responses in those particular design cells (this is reflected in the wider confidence intervals for the 0.9 error RT quantiles).

**Figure 6:**
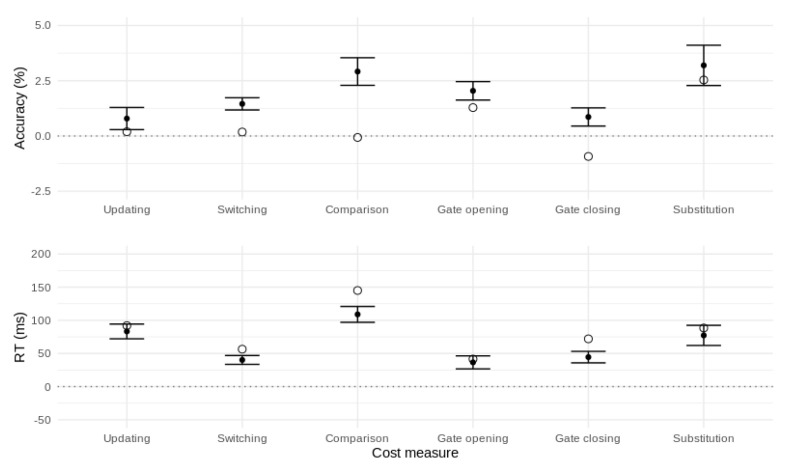
Fits of top model to reference-back cost effects. Unfilled circles represent data effects. Filled circles represent posterior model predictions with 95% credible intervals.

To further explore how well the model predicts specific reference-back cost effects, we calculated each cost effect on the models’ posterior predictions and compared them to the corresponding empirical effects. As shown in Figure 6, for the updating and substitution effects, the model predicts both accuracy and RT costs very closely. For the switching, comparison, and gate-closing effects, the model tends to predict a larger accuracy and smaller RT cost than was observed. For gate opening, the model predicts a slightly larger accuracy cost than was observed and fits the RT cost very closely. Overall, the model reproduces the pattern of RT costs closely, with some misfit of accuracy costs for switching, comparison, and gate closing.

### 3.4. Parameter Effects

**Figure 7:**
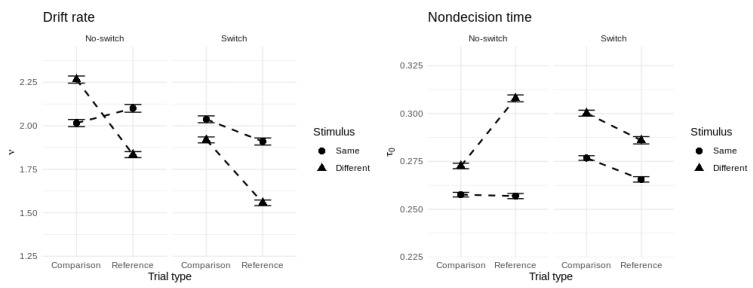
Group-averaged drift rate (**left panel**) and non-decision time (**right panel**) for each cell of the experimental design. Error bars represent ±1 posterior standard deviation.

**Figure A1:**
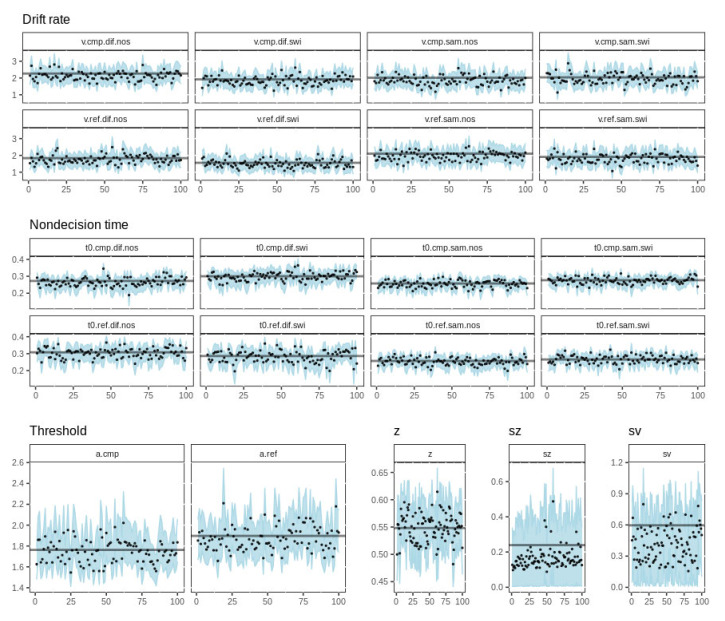
Parameter recovery for the top model. Each panel shows the recovered parameter values for 100 synthetic subjects (black dots) plotted against the known data-generating value (horizontal lines). The shaded region represents the 95% credible interval surrounding the recovered value. Note: *z* = starting point, *sz* = starting point variability, *sv* = drift rate variability.

In order to give us confidence in drawing inferences from the model’s parameters, we performed a parameter recovery study in which we assessed whether the model could recover the values of known data-generating parameters from simulated data in a precise and unbiased manner. Details of the simulation procedure and a visualization of the results are presented in Appendix C (Figure A1). Crucially, the parameters most relevant to our current theoretical questions—drift rate, non-decision time, and threshold—were each recovered closely and without bias. Figure 7 shows the group-averaged drift rate and non-decision time for each cell of the experimental design.

To draw inferences about the direction and magnitude of parameter effects in relation to the WM control processes measured by the reference-back task, we computed posterior distributions for each of the canonical cost measures in terms of the group-level posterior parameter samples. For example, taking the difference between repeated reference and comparison trials for a given parameter for each posterior sample gives the posterior probability distribution of the updating effect. For each effect distribution, we report a Bayesian *p*-value [57] that indicates the one-tailed probability that the effect does not run in the most sampled direction. To quantify the magnitude of each effect, we report the standardized difference between parameters (i.e., *Z* = *M/SD* of the effect distribution).

**Figure 8:**
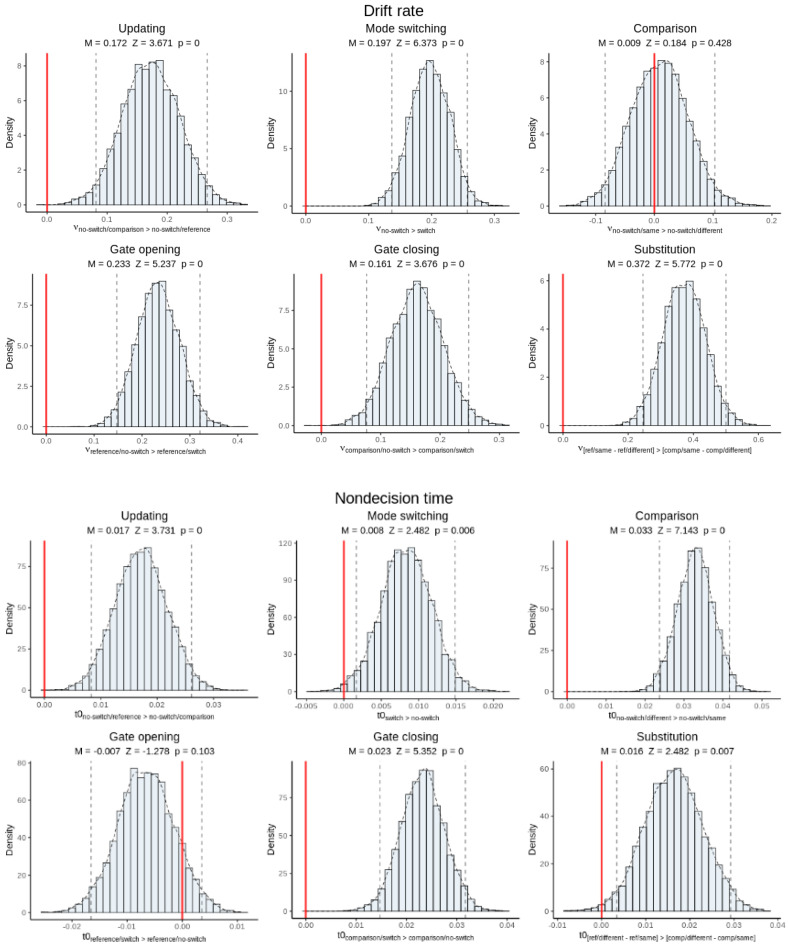
Posterior inference on canonical cost effects for hyper-mean drift rate (**top panels**) and non-decision time parameters (**bottom panels**). Each panel shows a histogram of the posterior distribution as well as the posterior mean, *Z-*score, and *p*-value of the effect. Dotted lines represent 95% credible intervals. Red lines indicate the zero point. Visually, if the red line appears outside of the credible interval, then the effect is significant. The *x*-axis labels state how the contrast is calculated.

Figure 8 shows histograms of the group-level posterior distributions for hyper-mean drift rate and hyper-mean non-decision time, with posterior means, *Z-*scores, and *p*-values, for each cost effect. All drift rate effects except for comparison type were significant, meaning that each reference-back cost measure was associated with poorer-quality evidence accumulation. All cost measures except for gate opening were also associated with longer non-decision times. Taken together, this suggests that the various behavioral costs measured by the reference-back task are driven both by additional WM control processes that run outside of the decision stage and by a reduction in the quality of information processing.

**Figure 9:**
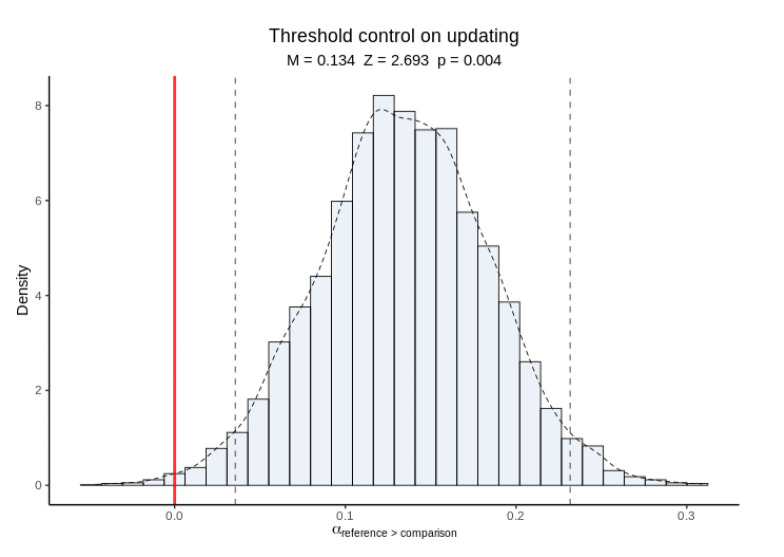
Posterior inference on hyper-mean thresholds in reference versus comparison trials. Raised thresholds in reference trials suggest cognitive control in response to the updating cue.

Posterior inference on hyper-mean thresholds indicated that thresholds were higher in reference trials than in comparison trials (Figure 9). This is consistent with subjects exerting cognitive control over thresholds in response to the updating cue in order to respond more cautiously when in updating mode.

### 3.5. Individual Differences

**Figure A2:**
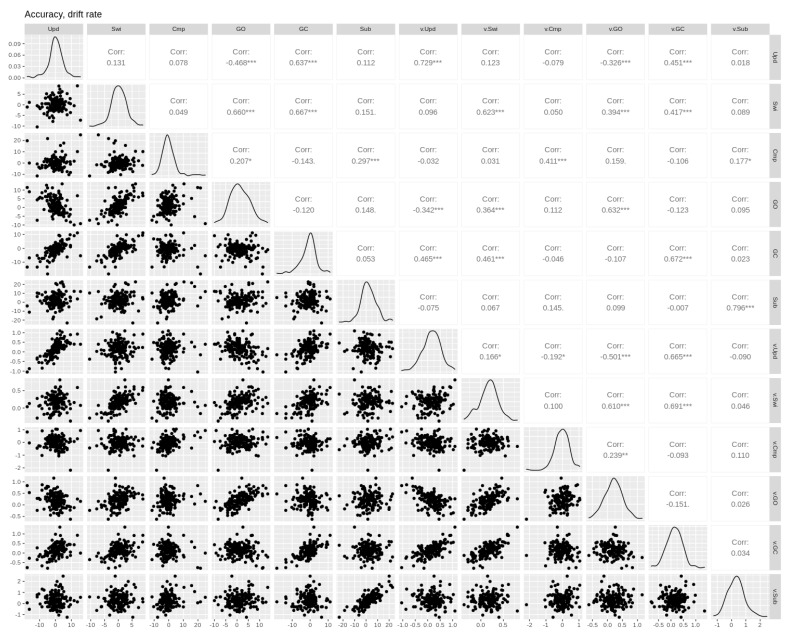
Pairwise Pearson correlations between drift rate and accuracy cost effects. Empirical effects are shown in the first six columns. Parameter effects are shown in the last six columns (prefixed with the parameter name). Upd = updating, Swi = switching, Cmp = comparison, GO = gate opening, GC = gate closing, Sub = substitution. Asterisks *, **, and *** denote *p*-values of <0.05, <0.01, and <0.001, respectively.

**Figure A3:**
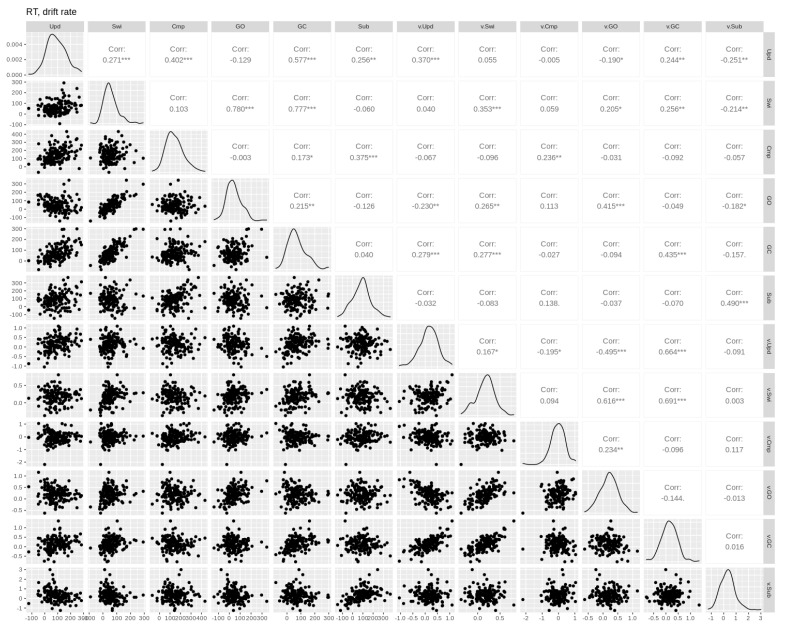
Pairwise Pearson correlations between drift rate and RT cost effects. Empirical effects are shown in the first six columns. Parameter effects are shown in the last six columns (prefixed with the parameter name). Upd = updating, Swi = switching, Cmp = comparison, GO = gate opening, GC = gate closing, Sub = substitution. Asterisks *, **, and *** denote *p*-values of <0.05, <0.01, and <0.001, respectively.

**Figure A4:**
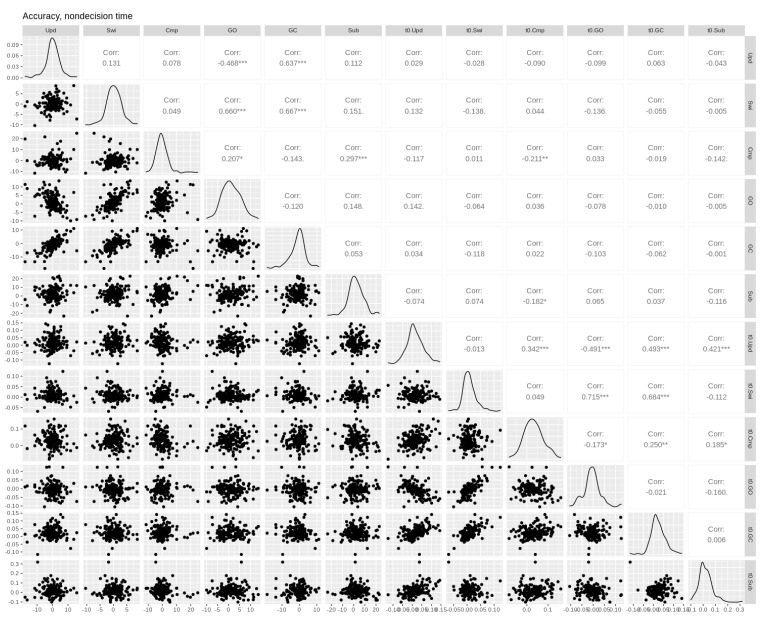
Pairwise Pearson correlations between non-decision time and accuracy cost effects. Empirical effects are shown in the first six columns. Parameter effects are shown in the last six columns (prefixed with the parameter name). Upd = updating, Swi = switching, Cmp = comparison, GO = gate opening, GC = gate closing, Sub = substitution. Asterisks *, **, and *** denote *p*-values of <0.05, <0.01, and <0.001, respectively.

**Figure A5:**
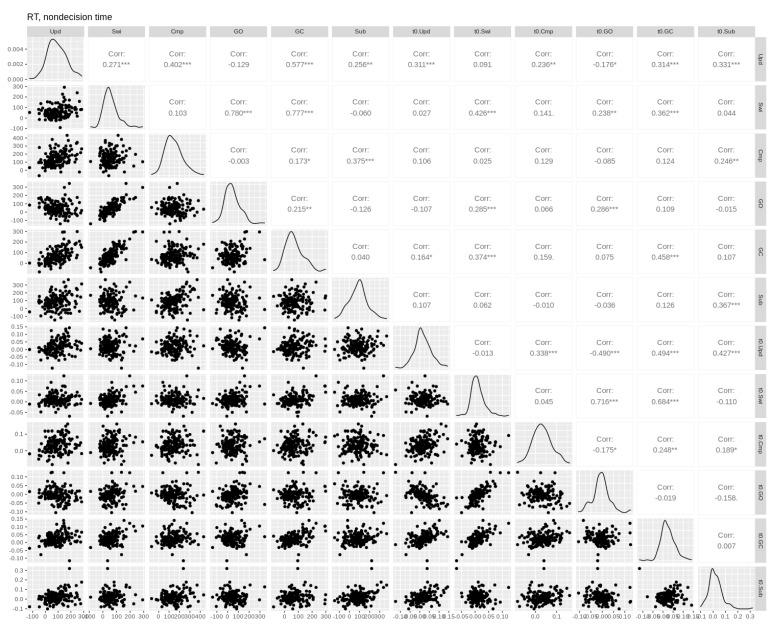
Pairwise Pearson correlations between non-decision time and RT cost effects. Empirical effects are shown in the first six columns. Parameter effects are shown in the last six columns (prefixed with the parameter name). Upd = updating, Swi = switching, Cmp = comparison, GO = gate opening, GC = gate closing, Sub = substitution. Asterisks *, **, and *** denote *p*-values of <0.05, <0.01, and <0.001, respectively.

**Figure A6:**
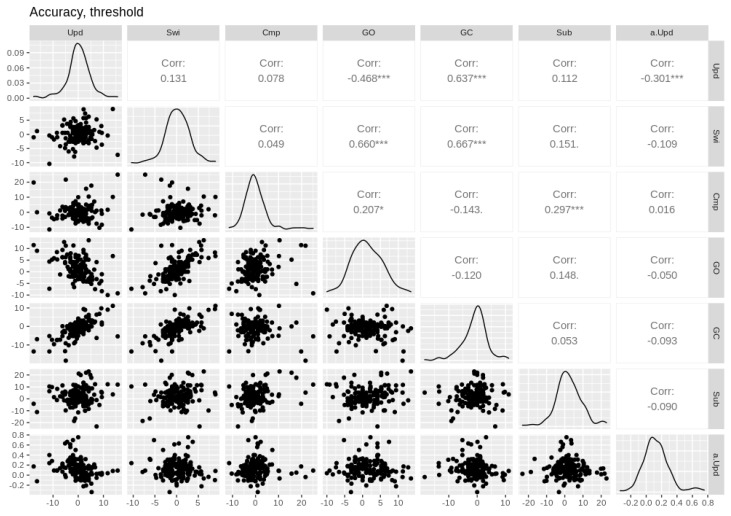
Pairwise Pearson correlations between threshold and accuracy cost effects. Empirical effects are shown in the first six columns. Parameter effects are shown in the last column (prefixed with the parameter name). Upd = updating, Swi = switching, Cmp = comparison, GO = gate opening, GC = gate closing, Sub = substitution. Asterisks * and *** denote *p*-values of <0.05, <0.01, and <0.001, respectively.

**Figure A7:**
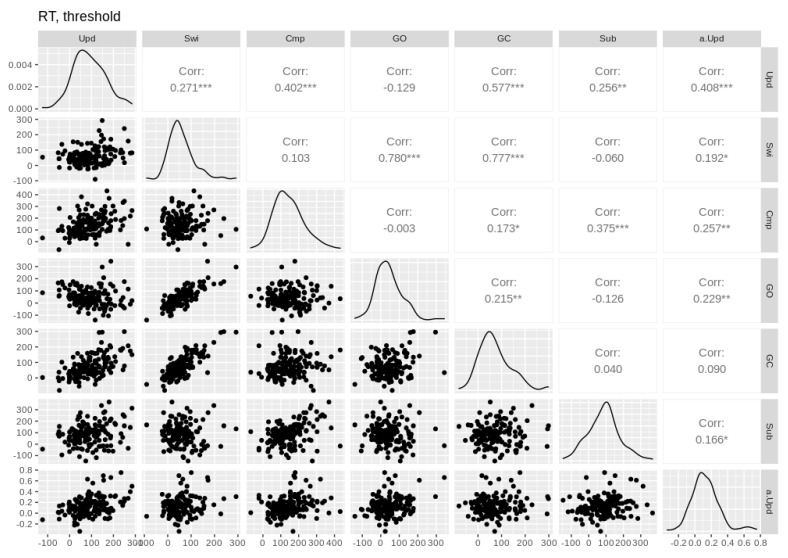
Pairwise Pearson correlations between threshold and RT cost effects. Empirical effects are shown in the first six columns. Parameter effects are shown in the last column (prefixed with the parameter name). Upd = updating, Swi = switching, Cmp = comparison, GO = gate opening, GC = gate closing, Sub = substitution. Asterisks *, **, and *** denote *p*-values of <0.05, <0.01, and <0.001, respectively.

Capitalizing on our large sample, we conducted an exploratory individual differences analysis to assess the relationship between subject-level parameter effects and empirical performance costs. To this end, we examined the Pearson correlations between individuals’ RT and accuracy costs and the subject-level maximum a posteriori estimates of drift rate, non-decision time, and threshold effects from the non-hierarchical version of the top model (i.e., without shrinkage of subject-level parameters toward the group mean, which violates the assumption of independent observations required for correlations). These correlations are illustrated in Appendix D (Figure A2, Figure A3, Figure A4, Figure A5, Figure A6 and Figure A7). Two outlying values of switching and comparison costs were excluded to avoid obtaining spuriously inflated correlations involving these variables.

All drift rate effects were correlated with their corresponding accuracy (*r* = [0.41–0.80]) and RT costs (*r* = [0.24–0.49]), whereas non-decision time effects were correlated with their corresponding RT effects only (*r* = [0.29–0.46]), except for a significant comparison effect for accuracy and a nonsignificant comparison effect for RT). This general pattern is to be expected, since changes in non-decision time cannot affect accuracy, whereas drift rates affect both RT and accuracy. The magnitude of threshold shifts between reference and comparison trials was positively correlated with RT costs of updating (*r* = 0.41) and negatively correlated with accuracy costs of updating (*r* = –0.30), which is also consistent with the function of thresholds in controlling trade-offs between speed and accuracy. Finally, we note that related parameter effects tended to correlate with each other. For example, gate opening and closing costs were correlated with updating and the more general switching cost for both drift rate and non-decision time (*r* = [−0.48–0.73]). Similarly, substitution costs were correlated with the more general updating cost for non-decision time (*r* = 0.45). However, drift rate and non-decision time effects were mostly uncorrelated with each other, indicating that these parameters accounted for distinct components of the observed effects. Overall, this gives us further confidence that our model is capturing this complex set of empirical effects—at both the group and individual levels—in a coherent and psychologically plausible manner.

## 4. Discussion

In this study, we performed the first detailed model-based analysis of behavioral costs in the reference-back task, which are assumed to reflect a set of cognitive control processes that support WM. Our data replicated the set of behavioral effects found in previous reference-back studies [11,12,14,21,22,23,24,25], including the asymmetrical costs of opening and closing the gate to WM [7,11,12,14,21]. The DDM provided close fits to empirical choice-RT distributions for each design cell of the reference-back, and model selection indicated that, for most subjects, drift rate and non-decision time each played an important role in explaining the observed differences in accuracy and RT. The model also provided a coherent account of individual differences in reference-back performance.

Posterior inference on model parameters indicated that updating, gating, and substitution costs were partly due to additional WM control processes running outside of the decision stage (a non-decision time effect) and partly due to a reduction in the quality of information processing (a drift rate effect). This latter finding is intriguing because current theories of WM updating assume that updating and gating are non-decision time processes that run outside of the decision-making stage and are thus not expected to interfere with the speed with which WM information is processed (i.e., drift rate). A potential explanation for this is that the same cascade of processes responsible for engaging WM control operations (e.g., updating and gating) also involves inhibition of the prepotent response—by slowing the rate of evidence accumulation—to allow more time for active control processes to finish. This is in line with EEG work with the reference-back task suggesting that oscillatory signals related to conflict monitoring are involved in triggering WM control processes when the need to use them arises [23,24]. Given that in our design the requisite WM control operations could not be predicted prior to trial onset, our inhibitory effects are an example of reactive control [30]. Reactive control processes operate automatically when triggered by inputs signaling the critical event (i.e., when the cognitive system detects a stimulus/response conflict or the need to update or open/close the gate). Event-triggered reactive inhibition of prepotent response drift rates has been reported in similar evidence accumulation modeling of event-based prospective memory, in which ongoing task drift rates were inhibited on trials containing an unexpected cue to perform a deferred action [31,32,33], Our present results are consistent with this work. In sum, our modeling suggests that performance costs on the reference-back task are due to a combination of reactive response inhibition that is triggered alongside WM control processes and the time taken for those processes to run outside of the decision stage.

In addition, our best model indicated that individuals set higher thresholds in reference trials (i.e., when cued to update WM) than in comparison trials (i.e., when cued to maintain/not update). At a strategic level, this increases the amount of evidence required to trigger a decision and thus reduces the likelihood of making premature responses based on outdated information (e.g., before the updating process has been completed). This highlights a further way in which individuals apply cognitive control to meet the demands of the reference-back paradigm.

In terms of neurocomputational theory, our findings are consistent with prominent neural network-based process models of WM, such as the prefrontal cortex–basal ganglia working memory (PBWM) model [16,17,18], in which the basal ganglia controls WM updating via a dynamic gating mechanism. In the PBWM, the prefrontal cortex holds WM representations (via recurrent excitation) in a default ‘gate-closed’ state of active maintenance against interference. The role of the basal ganglia is to learn [58], via dopamine-driven reinforcement learning [59], when to open the gate or ‘release the brakes’ on WM and thereby allow prefrontal representations to be rapidly updated in line with task goals [17]. In our modeling, this gate-opening mechanism was associated only with inhibited drift rates and not with prolonged non-decision time, which suggests that gate opening adds minimal time outside of the decision stage and that associated performance costs are driven primarily by response inhibition, triggered by the same cascade of processes as gating. However, we note that since gate opening and closing both (necessarily) involve switching between trial types, these measures may include effects related to task switching, such as task-set reconfiguration and proactive interference from previously active sets (we note that our updating and comparison measures were based only on no-switch trials and so were not contaminated by potential switching effects [4,60,61]). This would go some way toward explaining why both gating measures (and switching) were associated with lower drift rates, since interference from previously active sets and noise in the retrieved set would be expected to reduce the quality of decision processing on switch trials [62]. We also note that the larger costs associated with closing versus opening the gate (i.e., when switching to maintenance mode) provide further support for the PBWM’s assumption that WM sits in maintenance (gate-closed) mode by default. This is due to the common finding in the task switching literature that switching to an easier task takes longer than switching to a more difficult task [63,64]. Assuming that maintenance is less demanding than updating, as our drift rate and choice-RT effects suggest, then maintenance should be the default operating state and consequently should attract a larger switch cost. Clever experimental design, combined with stronger links to neural data, will be needed to further disentangle gating and switching processes and provide a clearer picture of the basal ganglia’s role in triggering such processes.

Our finding that individuals apply different threshold (response caution) settings when switching between updating and maintenance mode is consistent with the role of ‘top-down’ cortical control in the PBWM framework. However, because our model does not contain a learning mechanism, it does not explain how individuals acquire the cognitive control settings (and timing thereof) that they use to meet task demands. Future work may thus profit from integrating a PBWM-like learning mechanism into our evidence accumulation framework to obtain finer control over the temporal dynamics of reference-back performance (e.g., by having threshold and/or reactive control settings vary from trial to trial as a function of learning; see [65,66] for examples of such an approach in the domain of instrumental learning). In addition, we speculate that some of the minor misfits (e.g., to empirical switching and comparison costs) of our model were likely due to certain sequential or ‘carry-over’ effects that are unaccounted for in the current framework, such as proactive interference, priming, task-set inertia/reconfiguration, and Gratton effects arising from previously encountered stimuli and responses [11,42,43,62,67,68,69,70,71,72]. Due to our limited number of trials per subject, we were unable to conduct a thorough model-based analysis of sequential effects in the reference-back task. However, it has been suggested that tasks involving WM updating are particularly prone to interference from such sources, because frequent updating prevents the strong binding of items to locations in WM [5]. Incorporating dynamic adjustments of the cognitive system via trial-to-trial learning in the manner suggested may hold some promise in capturing these additional sources of variability in reference-back performance.

In terms of methodological advancement, our model-based approach offers a finer decomposition of reference-back performance costs in terms of latent decision processes than has previously been available. This is important because there is a lack of strong behavioral predictions from existing models of WM, and most models are silent on the specific WM control processes we seek to understand. Looking forward, we believe our approach will offer stronger links between theory and data in future work with the reference-back paradigm. For example, an obvious next step would be to use the model as a way to interpret behavioral and neural reference-back data through the same cognitive theory, as is done in model-based cognitive neuroscience (e.g., the ‘model-in-the-middle’ approach [73,74,75,76] and joint neural-behavioral modeling [35,77,78]). A string of recent work has adopted the reference-back task in the hope of understanding the neural basis of WM control processes [21,22,23,24] and the role of dopamine systems in controlling gating and updating [21,25] (see also [79,80]). Our approach to decomposing performance costs into latent cognitive processes may reveal links between brain and behavior that are masked at the level of accuracy and mean RT, and which would thus be missed in conventional analyses. We believe this kind of modeling will play a substantial role in identifying the neural substrates of WM control processes and furthering our understanding of WM more broadly. An interesting question for future joint neural-behavioral modeling of the reference-back task is to ask whether our reactive inhibition effects relate to activity in brain regions previously linked to reactive control (e.g., the lateral prefrontal cortex, ref. [30]).

One limitation of the current approach is that probe-referent decisions and WM control operations are both triggered concurrently at stimulus onset and are thus somewhat confounded in terms of our ability to measure them with the standard DDM. In our modeling, we assumed that the duration of WM operations was captured in the non-decision time parameter. Although it is useful in decomposing the loci of RT costs, non-decision time is by no means an explicit mechanism or a pure measure of any WM or cognitive control process elicited by the reference-back task. In short, we measured the effects of WM control operations on DDM parameters but did not explicitly model those operations. Future model development may explore options for integrating explicit WM mechanisms (e.g., updating, gating, switching) into an evidence accumulation framework. This would ultimately enable inferences to be drawn about target processes directly rather than via their secondary effects on decision-making processes.

A further consideration is the extent to which our results are robust to changes in the evidence accumulation model architecture. Our starting point was the standard DDM, in which drift rates represent the relative difference in evidence between the two possible choice options (i.e., same vs. different). However, reference-back decisions could also be modeled using race architectures (e.g., the linear ballistic accumulator [81] and racing Wald models [82]), in which evidence for competing options is accumulated independently and the overt response is determined by the first accumulator to reach threshold. When fit to the same data, the DDM and race architectures typically lead to qualitatively similar inferences (i.e., accumulation rate, threshold, and non-decision time effects in the DDM typically map onto accumulation rate, threshold, and non-decision time effects in independent race models, e.g., [83], but not always [84]). We leave it to future work to assess whether substantive differences arise between the DDM and race model architectures when applied to the reference-back paradigm.

## 5. Concluding Remarks

Overall, we believe our approach to modeling the reference-back paradigm holds promise in furthering our understanding of WM and the control processes that make such flexible decision-making possible. Our model provides the first comprehensive computational account of reference-back task performance and offers a coherent explanation of group- and individual-level empirical effects in terms of non-decision time, reactive inhibitory control, and threshold control when updating. We look forward to combining our analysis approach with neurophysiological measures to gain further insight into the neural basis of WM control processes.

## Figures and Tables

**Table 1:** Cost measures derived from the reference-back task.

Measure	Derivation	Interpretation
Updating	Difference between no-switch/reference and no-switch/comparison trials	Cost of updating WM
Comparison	Difference between no-switch/same (probe-referent match) and no-switch/different (probe-referent mismatch) trials	Cost of a mismatch between the probe stimulus and the WM referent
Switching	Difference between switch and no-switch trials	Cost of switching between WM modes
Gate opening	Difference between reference/switch and reference/no-switch trials	Cost specific to opening the gate to WM
Gate closing	Difference between comparison/switch and comparison/no-switch trials	Cost specific to closing the gate to WM
Substitution	Interaction of updating and comparison factors; difference between the cost of updating a new/mismatching item into WM and the cost of responding to a mismatching item without updating; (reference/different − reference/same) − (comparison/different − comparison/same)	Cost of updating a new item into WM

**Table 2:** Model selection.

Model	Parameters	DIC Difference from Top Model	*n*	%
Top model	21	0	56	37.3
Threshold fixed	20	179	46	30.7
Drift rate fixed	14	880	35	23.3
Non-decision time fixed	14	2720	13	8.7

The *n* and % columns denote the number and percentage of participants for whom each model was preferred (i.e., had the lowest DIC).

## Data Availability

Data and analysis code available upon request.

## Appendix A

### Significance Tests of Behavioral Effects

**Table A1:** Significance testing of accuracy effects using a generalized linear model with a probit link function.

Effect	*SS*	*df_effect_*	*Residual SS*	*df_within_*	*F*	*p*
Intercept	996.33	1	9.59	149	15,485.87	<0.001
U	0.05	1	0.32	149	23.48	<0.001
C	0.03	1	0.86	149	4.91	0.028
S	0.00	1	0.26	149	0.54	0.466
U × C	0.05	1	0.61	149	11.80	<0.001
U × S	0.04	1	0.33	149	16.28	<0.001
C × S	0.03	1	0.22	149	22.59	<0.001
U × C × S	0.00	1	0.27	149	1.54	0.217

U = updating (reference, comparison), C = comparison (same, different), S = switching (switch, no-switch).

**Table A2:** Significance testing of mean RT effects using a general linear model with a Gaussian link function.

Effect	*SS*	*df_effect_*	*Residual SS*	*df_within_*	*F*	*p*
Intercept	623.25	1	33.94	149	2736.57	<0.001
U	1.81	1	1.07	149	253.14	<0.001
C	7.34	1	2.27	149	482.69	<0.001
S	1.04	1	0.89	149	173.75	<0.001
U × C	0.60	1	0.68	149	131.42	<0.001
U × S	0.06	1	0.57	149	14.44	<0.001
C × S	0.04	1	0.36	149	18.29	<0.001
U × C × S	0.14	1	0.49	149	41.28	<0.001

U = updating (reference, comparison), C = comparison (same, different), S = switching (switch, no-switch).

## Appendix B

### Appendix B.1. Sampling

Posterior distributions for DDM parameters were estimated using the differential evolution Markov chain-Monte Carlo (DE-MCMC) sampling algorithm [85], which is more adept than conventional samplers at handling the high parameter correlations that often occur in sequential sampling models. For each model, the number of sampling chains was set at three times the number of parameters in the model. To remove the effects of autocorrelation between successive samples, chains were thinned by 10, meaning that every 10th iteration was kept. Sampling continued until a multivariate potential scale reduction factor [86,87] less than 1.1 was obtained, indicating that sampling chains were well-mixed, stationary, and converged. For the top model, we retained 6300 samples (63 chains × 100 iterations) of each parameter’s posterior distribution for each subject.

### Appendix B.2. Priors

Since this task has never been modeled before, we placed relatively broad and noninformative priors on model parameters. All drift rates were given a truncated normal prior with a mean and standard deviation of 1 (truncated at −10 and 10). All non-decision times were given a truncated normal prior with a mean and standard deviation of 0.2 (truncated at 0.05 and 1). Thresholds were given a truncated normal prior with a mean of 1.5 and standard deviation of 1 (truncated at 0 and 10). Starting point was assigned a truncated normal prior with a mean and standard deviation of 1 (truncated at 0 and 10). Starting point variability and drift rate variability were each assigned a truncated normal prior with a mean and standard deviation of 0.1 (truncated at 0 and 10). In the fully hierarchical model, each of these parameters was given a hyper (group-level) mean and standard deviation. Hyper means were assigned truncated normal priors with means and standard deviations as above. Hyper standard deviations were given gamma priors with shape and scale parameters equal to 1.

## Appendix C

### Parameter Recovery

We performed a parameter recovery study to assess the model’s ability to recover known parameter values. In this procedure, we first used the top model with known (i.e., maximum a posteriori) parameter values to simulate data for 100 synthetic subjects. We then fit the model to the simulated data and examined how closely the 100 sets of recovered parameters matched the known data-generating values. Figure A1 shows parameter recovery for the top model. Recovered values for the drift rate and non-decision time parameters were tightly clustered around the true value, with no clear tendency to over- or underestimate the true value. This gives us confidence that the model recovers these parameters accurately and without bias. Thresholds were also recovered closely and without bias, as was the starting point parameter. For the two variability parameters—*sz* and *sv*—recovered values tended to underestimate the data-generating value, although the true value was still enclosed well inside the 95% credible region. Given that these parameters were not of theoretical interest and can be held fixed without affecting our inferences from the model (at the expense of a higher DIC value), this small bias was not a concern in the present analyses.
